# Effects of purified anthocyanin supplementation on platelet chemokines in hypocholesterolemic individuals: a randomized controlled trial

**DOI:** 10.1186/s12986-016-0146-2

**Published:** 2016-11-25

**Authors:** Xiandan Zhang, Yanna Zhu, Fenglin Song, Yanling Yao, Fuli Ya, Dan Li, Wenhua Ling, Yan Yang

**Affiliations:** 1 Department of Nutrition, School of Public Health, Sun Yat-sen University (Northern Campus), Guangzhou, People’s Republic of China; 2 Guangdong Provincial Key Laboratory of Food, Nutrition and Health, Guangzhou, People’s Republic of China; 3 Department of Maternal and Child Health, School of Public Health, Sun Yat-sen University (Northern Campus), Guangzhou, People’s Republic of China; 4 School of Food Science, Guangdong Pharmaceutical University, Guangzhou, People’s Republic of China

**Keywords:** Anthocyanins, Platelet chemokines, Hypercholesterolemia, Inflammation, Serum lipids, CXCL7

## Abstract

**Background:**

It is becoming increasingly evident that platelet chemokines are involved in distinct aspects of atherosclerosis. The aim of this study was to examine the effects of long-term supplementation with purified anthocyanins on platelet chemokines in hypercholesterolemic individuals and to identify correlations of decreased platelet chemokine levels with serum lipid and inflammatory marker levels.

**Methods:**

A total of 146 hypercholesterolemic individuals were recruited and treated with 320 mg of purified anthocyanins (*n* = 73) or a placebo (*n* = 73) daily for 24 weeks in this randomized, double-blind, placebo-controlled trial.

**Results:**

Anthocyanin supplementation for 24 weeks significantly decreased the plasma CXCL7 (–12.32% vs. 4.22%, *P* = 0.001), CXCL5 (–9.95% vs. 1.93%, *P* = 0.011), CXCL8 (–6.07% vs. 0.66%, *P* = 0.004), CXCL12 (–8.11% vs. 5.43%, *P* = 0.023) and CCL2 levels (–11.63% vs. 12.84%, *P* = 0.001) compared with the placebo. Interestingly, the decreases in the CXCL7 and CCL2 levels were both positively correlated with the decreases in the serum low-density lipoprotein-cholesterol (LDL-C), high-sensitivity C-reactive protein (hsCRP) and interleukin-1β (IL-1β) levels after anthocyanin supplementation for 24 weeks. The decrease in the CXCL8 level was negatively correlated with the increase in the how-density lipoprotein-cholesterol (HDL-C) level and was positively correlated with the decrease in the soluble P-selectin (sP-selectin) level in the anthocyanin group. In addition, a positive correlation was observed between the decreases in the CXCL12 and tumornecrosis factor-α (TNF-α) levels after anthocyanin supplementation. However, the plasma CXCL4L1, CXCL1, macrophage migration inhibitory factor (MIF) and human plasminogen activator inhibitor 1 (PAI-1) levels did not significantly change following anthocyanin supplementation.

**Conclusions:**

The present study supports the notion that platelet chemokines are promising targets of anthocyanins in the prevention of atherosclerosis.

**Trial registration:**

ChiCTR-TRC-08000240. Registered: 10 December 2008.

## Background

Atherosclerosis is a type of chronic inflammatory disease resulting from disordered lipid metabolism and a maladaptive inflammatory response [1, 2]. Hypercholesterolemia has been identified as an independent risk factor for atherosclerosis [3] that is mainly attributed to the modulation of macrophage and platelet biogenesis and activity in early atherosclerotic lesions [4]. Numerous studies have demonstrated that platelet chemokines play an important role in the pathogenesis of hypercholesterolemia-induced atherosclerotic disease [5–7]. These chemokines contribute to the recruitment of circulating leukocytes and progenitor cells to the site of injured endothelium, forming a surface on which platelets are further activated to enhance inflammation [8, 9]. In addition, platelet chemokines have a possible pathogenic role in interactions among platelets, oxidized low-density lipoproteins (ox-LDLs), and peripheral leukocytes [10, 11]. The most abundant platelet-derived chemokines include CXCL4 (platelet factor 4; PF4) and CXCL7 (neutrophil-activating peptide-2; NAP-2). Several other chemokines, such as CCL5 (regulated on activation in normal T-cell expressed and secreted; RANTES), CXCL5 (epithelial neutrophil-activating peptide; ENA-78), CXCL12 (stromal cell-derived factor 1; SDF-1α), CXCL1 (growth-regulated oncogene-alpha; GRO-α), CXCL8 (interleukin-8; IL-8), CCL2 (monocyte chemotactic protein-1; MCP-1), CXCL4L1 (PF4alt), macrophage migration inhibitory factor (MIF) and human plasminogen activator inhibitor 1 (PAI-1), are abundantly released or expressed by platelets but have been primarily identified in other cell types [12–15]. Emerging evidence indicates that the plasma levels of some platelet chemokines, such as CXCL4, CCL5, CXCL5, CXCL1, CCL2 and CXCL8, are increased in hypercholesterolemia [16–19]. Hence, reduction of platelet chemokine levels has already been suggested as a feasible approach for preventing and treating atherosclerosis [20].

Numerous epidemiological and medical anthropological investigations have suggested that anthocyanins play a protective role against atherosclerosis [21, 22]. Anthocyanins, a category of phenolic flavonoids, are abundant in various fruits, vegetables and beverages and are commonly consumed in the human diet [23–25]. Consumption of anthocyanins beneficially influences inflammatory disorders, the lipid profile, platelet activation and obesity-related disorders [26–29]. Our previous studies have shown that purified anthocyanin supplementation reduces the levels of inflammatory biological markers and improves serum lipids and endothelial function [30–32]. We have also found that anthocyanins significantly inhibit platelet activation, thrombosis, platelet hyperactivity [33, 34] and platelet granule secretion of molecules such as CCL5, β-TG and soluble P-selectin (sP-selectin), both in vitro and in vivo [35]. However, the effects of purified anthocyanin supplementation on most other platelet chemokines in patients with hypercholesterolemia have not yet been assessed.

Therefore, we designed this randomized, double-blind, placebo-controlled, 24-week trial to investigate the effects of long-term supplementation with purified anthocyanins on platelet chemokines in subjects with hypercholesterolemia. We also assessed whether decreased platelet chemokine levels were correlated with changes in serum lipids and inflammatory biological markers after treatment.

## Methods

### Subjects

A total of 150 hypercholesterolemic individuals were recruited for this trial between November 2008 and December 2010 in Guangzhou, Guangdong, China. The participants were recruited from physical examination centers at three hospitals and ranged in age from 40 to 65 years old, as previously described [32, 35]. Briefly, the clinical inclusion criterion for participation in the trial was a fasting total cholesterol level of between 5.17 mmol/L and 8.01 mmol/L (approximately 200 mg/dL to 310 mg/dL). Individuals with a history of cardiovascular disease (CVD), hypertension, diabetes mellitus, thyroid disorder, smoking or the use of any drug that could influence the measurement of lipid parameters, inflammatory markers, or chemokines were excluded from the study. The study was approved by the ethics committee of Sun Yat-sen University (the protocol number is No. 200802; the clinical trial number is ChiCTR-TRC-08000240), and written informed consent was obtained from all participants.

### Study design

This randomized, double-blind, placebo-controlled trial was carried out for 24 weeks. Briefly, the participants were randomly assigned to either an anthocyanin group (*n* = 75; 31 males and 44 females) or a placebo group (*n* = 75; 32 males and 43 females). During the trial period, the participants were instructed to consume two anthocyanin capsules or placebo capsules twice daily for 24 weeks. The anthocyanin capsules provided a total daily intake of 320 mg of anthocyanins. The participants were told not to change their dietary habits and not to consume any anthocyanin-rich foods or any other medications that might affect the serum anthocyanin level over the following 24 weeks. Fasting blood samples (≥10 h) were collected at the beginning and at weeks 12 and 24 of the trial.

### Materials and reagents

The anthocyanin and placebo capsules were obtained from Polyphenols AS (Sandnes, Norway). CXCL7 human ELISA kits were purchased from Abcam (Cambridge, UK), and human CXCL8 and CCL2 ELISA kits were purchased from eBioscience (San Diego, CA, USA). In addition, MILLIPLEX Multi-analyte Profiling (MAP) Human Cytokine/Chemokine Kits for human CXCL12, CXCL5 and PAI-1 were purchased from Millipore (Billerica, MA, USA). Further, human CXCL1 and MIF ELISA kits were obtained from R&D Systems (Minneapolis, MN, USA) and human CXCL4L1 ELISA kits were obtained from LifeSpan Biosciences (Denver, CO, USA).

### Biochemical analysis

Fasting blood samples were collected at baseline and weeks 12 and 24. Blood samples were drawn into heparin anticoagulant tubes for all study participants early in the morning after they had fasted overnight for 12 h, and the samples were centrifuged at 1500 × g for 15 min at 4 °C within 2 h to separate the plasma. Then, the plasma was stored at –80 °C until testing. The concentrations of serum lipids, including total cholesterol, triglycerides, low-density lipoprotein cholesterol (LDL-C) and high-density lipoprotein cholesterol (HDL-C), were measured enzymatically using an automatic analyzer. The serum high-sensitivity C-reactive protein (hsCRP) levels were determined using a Hitachi 911 automated assay analyzer and the immunoturbidimetric method. The serum tumornecrosis factor-α (TNF-α) level was measured using a commercially available ELISA kit obtained from R&D Systems (Minneapolis, MN, USA). Further, the plasma interleukin-1β (IL-1β) and sP-selectin levels were measured with commercial ELISA kits obtained from eBioscience (San Diego, CA, USA) as previously described [32, 35].

### Analysis of platelet chemokine levels

Platelet chemokines levels in the plasma were quantified using commercial ELISA kits or MILLIPLEX MAP Human Cytokine/Chemokine Kits. The samples used in ELISA were diluted based on the pre-experiment concentrations and were run in duplicate in accordance with the manufacturer's instructions. The recombinant products and standard solutions provided in the kits were used as positive controls. The optical density of each well was determined at 450 nm using a plate reader within 30 min. Average absorbance values were calculated for each set of duplicate standards and samples. The data were analyzed based on the standard curve values. Multiplex immune assays were performed according to the manufacturer's instructions.

### Statistical analysis

The platelet chemokine data were expressed as the mean ± SD or as the mean and 95% confidence interval (CI). The baseline characteristics of the two groups were compared using unpaired Student's *t*-test for continuous data. Further, repeated-measures ANCOVA was conducted to determine the effects of anthocyanin supplementation on platelet chemokines in the hypercholesterolemic subjects. The percentage changes in the platelet chemokine levels were calculated as follows: (value at week 24 – value at baseline)/value at baseline × 100. The average percentage change was expressed as the mean (95% CI). Differences in the average percentage changes in the platelet chemokine levels between the two groups were evaluated using unpaired Student's *t*-test. Comparisons of the absolute changes in the platelet chemokine levels after the 24-week intervention within the groups were conducted using paired Student's *t*-test. In addition, Pearson correlation coefficients (*r*) were calculated to assess the associations between the decreased plasma chemokine concentrations and the changes in the plasma lipid and inflammatory maker levels over the 24-week study period. Differences were considered significant at a *P* < 0.05. All statistical analyses were performed using IBM SPSS Statistics 20 (SPSS Inc., Chicago, IL, USA).

## Results

### Baseline characteristics of the subjects

A total of 4 participants withdrew from this trial, and 146 subjects ultimately completed it (*n* = 73 in the anthocyanin group; and *n* = 73 in the placebo group). The distributions of age, the anthropometric characteristics and the mean daily intake of nutrients were uniform between the two groups, as previously described (Table 1) [32]. No subjects reported any adverse events resulting from the treatment throughout the intervention period.

**Table 1:** Anthropometric characteristics and daily nutrient intake of the participants

	Placebo (*n* = 73)			Anthocyanins (*n* = 73)			*P*-value^a^
	Baseline	12 wk	24 wk	Baseline	12wk	24 wk	
Weight (kg)	70.1 ± 9.8	69.7 ± 9.9	69.5 ± 9.6	68.9 ± 8.8	67.8 ± 8.9	66.5 ± 8.1	0.342
BMI (kg/m2)	26.8 ± 2.0	26.7 ± 2.1	26.8 ± 2.2	26.4 ± 2.1	25.9 ± 2.0	26.1 ± 2.0	0.063
Waist circumference (cm)	89.6 ± 7.9	89.5 ± 8.2	89.2 ± 8.3	88.6 ± 6.4	87.4 ± 6.4	87.2 ± 6.5	0.226
Hip circumference (cm)	100.3 ± 6.3	100.3 ± 6.5	101.4 ± 6.4	100.0 ± 5.0	99.1 ± 5.2	98.3 ± 4.6	0.308
Waist/hip ratio	0.89 ± 0.06	0.89 ± 0.06	0.88 ± 0.05	0.89 ± 0.05	0.88 ± 0.05	0.88 ± 0.05	0.624
Systolic BP (mmHg)	124.3 ± 16.0	123.8 ± 15.0	120.7 ± 15.1	126.2 ± 14.9	119.5 ± 12.5	119.4 ± 12.6	0.135
Diastolic BP(mmHg)	82.8 ± 10.5	81.2 ± 9.1	81.1 ± 9.8	84.7 ± 10.7	82.8 ± 9.6	82.6 ± 9.3	0.219
Energy (kcal/d)	2163.6 ± 124.2	2168.2 ± 116.1	2154.4 ± 114.2	2185.4 ± 132.5	2199.2 ± 123.7	2171.6 ± 121.8	0.684
Protein (g/d)	84.8 ± 10.8	83.6 ± 9.4	81.4 ± 9.5	85.7 ± 10.5	84.5 ± 9.8	86.5 ± 10.9	0.702
% of energy	18.5 ± 3.1	18.4 ± 2.7	17.3 ± 3.0	18.6 ± 2.9	18.5 ± 3.1	19.1 ± 3.2	0.746
Total fat (g/d)	82.4 ± 18.2	80.1 ± 17.2	80.2 ± 16.8	80.2 ± 15.8	83.7 ± 17.6	84.9 ± 18.1	0.282
% of energy	26.5 ± 3.5	26.3 ± 3.2	24.3 ± 4.0	26.4 ± 3.2	27.4 ± 4.0	27.6 ± 4.3	0.493
Total carbohydrates (g/d)	258.6 ± 34.2	258.8 ± 40.5	261.0 ± 39.7	262.1 ± 43.8	263.2 ± 42.5	260.4 ± 40.3	0.161
% of energy	55.2 ± 4.7	55.3 ± 4.6	56.9 ± 4.5	55.0 ± 5.3	55.3 ± 4.8	54.2 ± 4.2	0.824
Cholesterol (mg/d)	339.4 ± 43.2	340.1 ± 38.4	331.3 ± 40.4	341.3 ± 40.2	342.5 ± 39.3	340.7 ± 43.6	0.327
Fiber (g/d)	20.5 ± 4.4	20.8 ± 4.2	19.2 ± 4.0	20.6 ± 5.0	20.6 ± 4.5	21.3 ± 4.7	0.644

The data are expressed as the mean ± SD

No significant differences in any variable were observed between the two groups at baseline, as determined using unpaired Student’s *t*-test

^a^The intervention had no significant effects on daily nutrient intake, as determined by repeated-measures ANOVA

### Effects of anthocyanin supplementation on platelet chemokines

The plasma platelet chemokine concentrations at baseline and at weeks 12 and 24 after the intervention are shown in Table 2. No significant differences in these concentrations were observed between the two groups at baseline. At week 12, only the plasma CXCL5 (*P* = 0.021) and CXCL8 concentrations (*P* = 0.015) were significantly decreased by anthocyanins compared with the baseline concentrations. At week 24, anthocyanin supplementation resulted in significant decreases in the plasma CXCL7 [–12.32% (95% CI, –19.25 to –5.00%), *P* = 0.001], CXCL5 [–9.95% (95% CI, –15.84 to –3.56%), *P* = 0.011], CXCL8 [–6.07% (95% CI, –8.15 to –4.23%), *P* = 0.004], CXCL12 [–8.11% (95% CI, –15.98 to –0.30%), *P* = 0.023] and CCL2 concentrations [–11.63% (95% CI, –18.16 to –4.85%), *P* = 0.001] compared with the placebo. Although the plasma CXCL4L1, CXCL1, MIF and PAI-1 concentrations also exhibited trends toward slight decreases during the treatment period, no significant changes were detected between the two groups.

**Table 2:** Changes in the platelet chemokine levels in the participants

	Placebo (*n* = 73)				Anthocyanins (*n* = 73)				*P-*value^b^
	Baseline	12 wk	24 wk	Mean change,% (95% CI)^a^	Baseline	12 wk	24 wk	Mean change,% (95% CI)^a^	
CXCL7 (ng/ml)	167.63 ± 38.78	165.82 ± 37.83	166.20 ± 31.92	4.22	170.52 ± 44.86	157.76 ± 43.21	138.83 ± 30.49^c^	−12.32	0.011
				(−2.33 to 11.05)				(−19.25 to −5.00)^d^	
CXCL5 (pg/ml)	159.43 ± 29.35	157.85 ± 31.80	157.32 ± 29.74	1.93	160.40 ± 36.26	150.78 ± 28.98 ^c^	137.61 ± 26.14^c^	−9.95	0.006
				(−4.18 to 9.28)				(−15.84 to −3.56)^d^	
CXCL8 (pg/ml)	22.97 ± 2.73	22.91 ± 2.51	22.97 ± 4.24	0.66	22.93 ± 1.96	22.32 ± 1.61 ^c^	21.40 ± 1.07^c^	−6.07	0.016
				(−2.99 to 4.88)				(−8.15 to −4.23)^d^	
CXCL12 (ng/mL)	2.16 ± 0.53	2.15 ± 0.51	2.14 ± 0.46	5.43	2.19 ± 0.56	2.11 ± 0.34	1.86 ± 0.47^c^	−8.11	0.020
				(−2.03 to 14.16)				(−15.98 to −0.30)^d^	
CCL2 (pg/mL)	504.99 ± 165.34	502.68 ± 157.33	515.76 ± 132.04	12.84	502.97 ± 139.91	476.96 ± 113.74	414.61 ± 87.14^c^	−11.63	0.014
				(1.05 to 27.23)				(−18.16 to −4.85)^d^	
CXCL4L1 (pg/ml)	124.29 ± 88.87	134.00 ± 69.82	135.16 ± 75.07	0.08	126.52 ± 67.05	126.44 ± 75.21	112.27 ± 86.78	−0.80	0.769
				(−0.21 to 0.35)				(−2.07 to 0.06)	
CXCL1 (ng/ml)	20.96 ± 3.15	21.94 ± 2.38	22.92 ± 3.67	0.07	21.30 ± 4.71	21.00 ± 3.37	21.07 ± 1.79	−0.02	0.529
				(−0.06 to 0.19)				(−0.24 to 0.16)	
PAI-1 (ng/ml)	101.52 ± 41.81	106.13 ± 48.55	108.84 ± 14.31	0.08	104.07 ± 52.84	107.16 ± 36.38	99.62 ± 42.35	−0.44	0.888
				(−0.10 to 0.29)				(−1.34 to 0.20)	
MIF (ng/ml)	32.91 ± 10.02	30.46 ± 9.54	29.51 ± 14.58	−9.90	32.69 ± 13.94	31.26 ± 8.53	26.93 ± 10.56	2.65	0.801
				(−25.96 to 7.13)				(−32.90 to 39.14)	

The data are expressed as the mean ± SD

No significant differences in any variable were observed between the two groups at baseline, as determined using unpaired Student’s *t*-test

^a^Calculated as (value at week 24 – value at baseline)/value at baseline × 100

^b^The effects of the intervention on these variables were evaluated by repeated-measures ANOVA

^c^ *P* < 0.05 vs. baseline, as assessed by paired Student’s *t*-test

^d^ *P* < 0.05 vs. percentage changes in the placebo group, as assessed by unpaired Student’s *t*-test

### Effects of anthocyanin supplementation on serum lipids

The serum lipid levels at baseline and 24 weeks are listed in Table 3, as previously described [32]. In the anthocyanin group, the HDL-C level significantly increased from 1.22 (0.23) mmol/L at baseline to 1.37 (0.22) mmol/L at week 24 (*P* = 0.018), accompanied by a significant reduction in the LDL-C level from 3.36 (0.58) mmol/L to 3.01 (0.41) mmol/L (*P* = 0.038). Moreover, significant differences in the HDL-C (*P* = 0.036) and LDL-C levels (*P* = 0.030) were detected between the two groups after 24 weeks.

**Table 3:** Changes in the lipid profiles of the participants

	Placebo (*n* = 73)			Anthocyanins (*n* = 73)			*P-*value^b^
	Baseline	24 wk	Mean change, % (95% CI)^a^	Baseline	24 wk	Mean change, % (95% CI)^a^	
Total cholesterol (mmol/L)	6.48 ± 0.84	6.25 ± 0.83	−3.6 (−7.8 to 0.6)	6.45 ± 1.02	6.18 ± 0.82	−2.9 (−6.3 to 0.5)	0.556
HDL-cholesterol (mmol/L)	1.24 ± 0.21	1.23 ± 0.20	−0.9 (−5.2 to 3.4)	1.22 ± 0.23	1.37 ± 0.22^c^	14.0 (7.9 to 20.2)^d^	0.036
LDL-cholesterol (mmol/L)	3.29 ± 0.47	3.30 ± 0.52	0.3 (−2.9 to 3.5)	3.36 ± 0.58	3.01 ± 0.41^c^	−10.4 (−14.8 to 6.0)^d^	0.030
Triacylglycerol (mmol/L)	2.41 (1.47 to 2.70)	2.34 (1.35 to 2.62)	−3.2 (−7.6 to 1.2)	2.45 (1.53 to 2.74)	2.35 (1.37 to 2.61)	−4.8 (−9.8 to 0.2)	0.462

The data are expressed as the mean ± SD

No significant differences in any variable were observed between the two groups at baseline, as determined using unpaired Student’s *t*-test

^a^ Calculated as (value at week 24 – value at baseline)/value at baseline × 100

^b^ The effects of the intervention on these variables were evaluated by repeated-measures ANOVA

^c^ *P* < 0.05 vs. baseline, as assessed by paired Student’s *t*-test

^d^ *P* < 0.05 vs. percentage changes in the placebo group, as assessed by unpaired Student’s *t*-test

### Effects of anthocyanin supplementation on serum inflammatory biomarkers

The serum levels of the inflammatory molecules hsCRP, TNF-α, IL-1β and sP-selectin in the participants at baseline and at weeks 12 and 24 are summarized in Table 4, as previously described [32, 35]. The hsCRP and IL-1β levels were reduced by anthocyanin supplementation at weeks 12 and 24, respectively, compared with the baseline levels. Further, anthocyanin supplementation for 24 weeks led to significant decreases in the plasma hsCRP [–21.6% (95% CI, –37.5 to –5.7%)], IL-1β [–12.8% (95% CI, –24.4 to –1.2%)], and sP-selectin levels [–5.9% (95% CI, –17.7 to 6.0%)] compared with the placebo.

**Table 4:** Changes in the inflammatory cytokine levels in the participants

	Placebo (*n* = 73)				Anthocyanin (*n* = 73)				*P-*value^b^
	Baseline	12 wk	24 wk	Mean change, % (95% CI)^a^	Baseline	12 wk	24 wk	Mean change, % (95% CI)^a^	
hsCRP (mg/L)	2.26	2.23	2.19	−2.5	2.25	1.95	1.74	−21.6	0.001
	(0.97 to 3.72)	(1.08 to 3.76)	(0.93 to 3.82)	(−7.0 to 2.1)	(1.06 to 4.25)	(0.92 to 2.84)^c^	(0.86 to 2.60)	(–37.5 to –5.7)^d^	
TNF-α (pg/mL)	18.0 ± 6.0	19.1 ± 6.7	18.5 ± 5.4	2.8 (−3.4 to 9.1)	18.7 ± 6.4	17.9 ± 5.1	18.4 ± 5.6	−1.6 (−5.6 to 3.4)	0.673
IL-1β (pg/mL)	4.77 ± 1.71	4.23 ± 0.91	4.71 ± 1.60	−1.3 (−5.3 to 2.7)	5.18 ± 2.11	4.62 ± 1.20^c^	4.51 ± 1.60^c^	−12.8 (−24.4 to −1.2)^d^	0.019
sP-selectin (ng/mL)	149.9 ± 42.7	139.9 ± 39.0	139.9 ± 39.0	−2.4 (−15.4 to 10.6)	150.9 ± 37.0	144.4 ± 35.3	134.6 ± 32.8	−5.9 (−17.7 to 6.0)^d^	0.027

The data are expressed as the mean ± SD

No significant differences in any variable were observed between the two groups at baseline, as determined using unpaired Student’s *t*-test

^a^ Calculated as (value at week 24 – value at baseline)/value at baseline × 100

^b^ The effects of the intervention on these variables were evaluated by repeated-measures ANOVA

^c^ *P* < 0.05 vs. baseline, as assessed by paired Student’s *t*-test

^d^ *P* < 0.05 vs. percentage changes in the placebo group, as assessed by unpaired Student’s *t*-test

### Correlations between alterations in plasma platelet chemokine and serum lipid levels

**Fig. 1:**
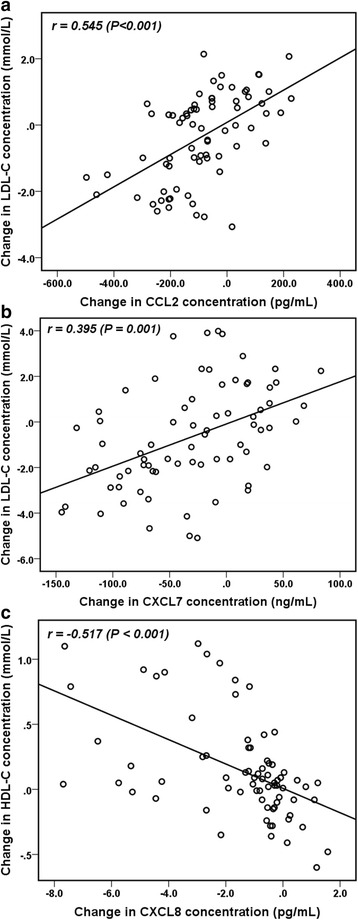
Significant correlations between changes in plasma platelet chemokine and serum lipid levels. After 24 weeks, correlations were detected between the changes in the plasma CCL2 (**a**) and CXCL7 levels (**b**) and the change in the LDL-C level, as well as between the changes in the plasma CXCL8 (**c**) and HDL-C levels, in the anthocyanin group. The data were evaluated using Pearson correlation coefficients (*r*)

After 24 weeks of anthocyanin supplementation, the changes in the plasma CCL2 (*r* = 0.545, *P* < 0.001) (Fig. 1a) and CXCL7 levels (*r* = 0.395, *P* = 0.001) (Fig. 1b) were positively correlated with the change in the LDL-C level. In addition, the change in the CXCL8 level (*r* = –0.517, *P* < 0.001) (Fig. 1c) was negatively correlated with the change in the HDL-C level in the anthocyanin group.

### Correlations between alterations in plasma platelet chemokine levels and serum inflammatory biomarker levels

**Fig. 2:**
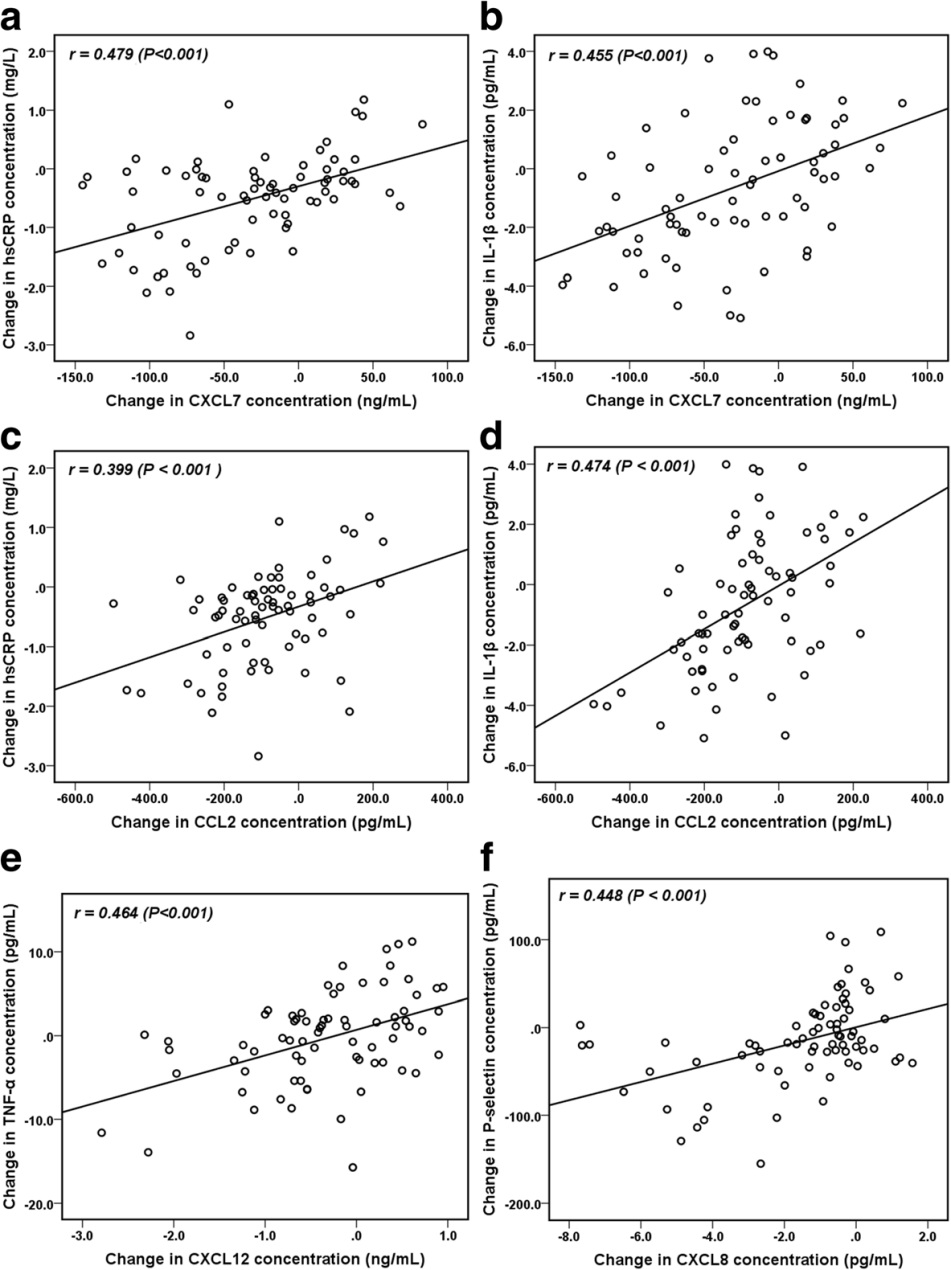
Significant correlations between changes in plasma platelet chemokine levels and inflammatory biomarker levels. After 24 weeks, correlations were detected between the change in the plasma CXCL7 level and the changes in the hsCRP (**a**) and IL-1β levels (**b**); between the change in the plasma CCL2 level and the changes in the hsCRP (**c**) and IL-1β levels (**d**); between the changes in the plasma CXCL12 and TNF-α levels (**e**); and between the changes in the plasma CXCL8 and sP-selectin levels (**f**) in the anthocyanin group. The data were evaluated using Pearson correlation coefficients (*r*)

After 24 weeks of anthocyanin intervention, the change in the plasma CXCL7 level exhibited a positive correlation with the changes in the hsCRP (*r* = 0.479, *P* < 0.001) (Fig. 2a) and IL-1β levels (*r* = 0.455, *P* < 0.001) (Fig. 2b). Interestingly, the change in the CCL2 level was also positively correlated with the changes in the hsCRP (*r* = 0.399, *P* < 0.001) (Fig. 2c) and IL-1β levels (*r* = 0.474, *P* < 0.001) (Fig. 2d). Furthermore, the change in the plasma CXCL12 level was significantly correlated with the change in the TNF-α level (*r* = 0.464, *P* < 0.001) (Fig. 2e). In addition, a positive correlation was detected between the changes in the CXCL8 and sP-selectin levels (*r* = 0.448, *P* < 0.001) (Fig. 2f) after anthocyanin intervention for 24 weeks.

## Discussion

Platelet chemokines are involved in inflammatory reactions, immune responses, and other aspects of the development of atherosclerosis [36]. In the present study, we first demonstrated that the plasma levels of the platelet chemokines CXCL7, CXCL5, CXCL8, CXCL12 and CCL2 were significantly decreased in hypercholesterolemic subjects after anthocyanin supplementation for 24 weeks. Furthermore, we found that the decreased levels of some platelet chemokines after anthocyanin treatment were closely correlated with the serum lipid and inflammatory molecule levels. These results indicated that anthocyanins exerted beneficial effects on the platelet chemokine levels, serum lipids and inflammatory factors, thereby inhibiting atherosclerosis.

Upon activation, platelets release a number of chemokines that may play important roles as first-line inflammatory mediators [37]. The most abundant platelet chemokine is CXCL7, which recruits and activates neutrophils at sites of vessel wall injury, inducing the inflammatory response within the atherosclerotic plaque [38]. CXCL5 has been shown to be abundantly produced and secreted by activated platelets, thereby attracting neutrophils, enhancing the cholesterol efflux capacity of macrophages and regulating foam cell formation [39–41]. CXCL12 plays an important role in leukocyte recruitment and neointima formation at sites of arterial injury [42]. Ahmed et al. have shown that epigallocatechin-3-gallate (EGCG) (10–50 μM), an active constituent in green tea, significantly inhibits the IL-1β-induced production of CXCL5, CCL5 and CXCL1 incubated with rheumatoid arthritis synovial fibroblasts in serum-free medium for 12 h [43]. Other polyphenols, such as wogonin [44], flavonoid naringenin [45] and quercetin [46], have been showed to inhibit CXCL5 in vitro. However, no study has examined the effects of anthocyanins on CXCL7, CXCL5 or CXCL12 to date. In the present study, we first showed that long-term supplementation with purified anthocyanins reduced the plasma levels of the platelet chemokines CXCL7, CXCL5 and CXCL12 in subjects with hypercholesterolemia. The role of anthocyanins in reducing platelet chemokine levels may contribute to their beneficial effects in hypercholesterolemic subjects.

In hypercholesterolemia, surface-adherent platelets express CXCL8 or CCL2 to recruit leukocytes, which adhere to the denuded artery and contribute to neointimal lesion formation [47, 48]. Recently, Karlsen et al. have demonstrated that the plasma CXCL8 level is decreased in healthy adults after supplementation with purified anthocyanins (300 mg/d) for 3 weeks [49]. Further, Kuntz et al. have revealed that treatment with an anthocyanin-rich grape extract for 4 h significantly attenuates CXCL8 secretion in activated human umbilical vein endothelial cells (HUVECs) [50]. In addition, administration of an anthocyanin-rich black elderberry extract (13% anthocyanins) for 10 weeks has been shown to significantly lower the serum chemokine CCL2 level in hyperlipidemic mice compared with control mice [51]. Garcia-Alonso et al. have shown that the circulating plasma CCL2 level is decreased in healthy volunteers after treatment with 12 g anthocyanin extract from red wine for 24 h [52]. Moreover, Björk et al. have found that CCL2 secretion by human primary fat cells is suppressed following treatment with the anthocyanin cyanidin-3-glucoside (130 nM) for 48 h [53]. In contrast, another study conducted by Kuntz et al. has revealed that in healthy young female volunteers, the CXCL8 and CCL2 levels are not affected by consumption of 330 ml/d of anthocyanin-rich fruit beverages for 14 days [54]. These inconsistencies among studies may be related to differences in the dose of anthocyanins used, the duration of the intervention and the study subjects. Our study is the first to show the long-term effects of purified anthocyanins on CXCL8 and CCL2 in Chinese individuals with hypercholesterolemia. These results represent novel, potentially valuable evidence supporting the beneficial effects of anthocyanins on CXCL8 and CCL2.

In addition to the above mentioned platelet chemokines, platelets also secrete CXCL1, CXCL4L1, MIF and PAI-1, which are crucially involved in interactions with platelets, leukocytes and endothelial cells during the early stage of atherogenesis [55, 56]. In an ApoE*3Leiden mouse model, administration of an anthocyanin-rich extract, Mirtoselect (the original standardized bilberry extract), for 20 weeks has been shown to attenuate the high-cholesterol diet-induced increase in the CXCL1 level [57]. Similarly, Jiang et al. have found that administration of anthocyanins from purple sweet potato and purple potato (5 mg/kg) to Kunming mice for 5 weeks inhibits the increased CXCL1 mRNA expression induced by alcohol [58]. Another animal study has revealed that administration of ≥5% Saskatoon berry powder to leptin receptor-deficient mice for 4 weeks suppresses the increase in the PAI-1 level [59]. Further, Lamy et al. have shown that delphinidin down-regulates the PAI-1 level in glioblastoma cells [60]. Broekhuizen et al. have found that the MIF and CCL2 levels are reduced following consumption of a polyphenol-rich extract (500 mg daily) for 4 weeks in subjects with clustered cardiometabolic risk factors [61]. In contrast with the above results, another study has shown that administration of an anthocyanin-rich black elderberry extract (0.17% anthocyanin, w/w) for 16 weeks does not decrease the serum PAI-1 level in diet-induced obese mice [62]. However, no studies have examined the effects of purified anthocyanins on these platelet chemokines in individuals with hypercholesterolemia. In our study, the CXCL1, CXCL4L1, MIF and PAI-1 levels were not significantly altered after anthocyanin supplementation for 24 weeks, although slight decreases were observed. Therefore, we speculate that these chemokines may not be sensitive to anthocyanins in hypercholesterolemic patients or that these patients may require a larger intervention dose or a longer intervention period to observe the significant inhibitory effects of anthocyanins.

Hypercholesterolemia is associated with elevated levels of peripheral leukocytes, platelets and cholesterol-induced pro-inflammatory cytokines [63]. Evidence suggests that platelet chemokines are involved in interactions between ox-LDL, platelets and leukocytes, possible contributing to atherogenesis in hypercholesterolemia [64, 65]. The primary platelet chemokines CXCL4 and CCL2 have been demonstrated to co-localize with ox-LDL in atherosclerotic lesions and to facilitate ox-LDL uptake by monocytes [66, 67]. In addition, platelet chemokines are produced following stimulation of TNF-α and IL-1β [68], which result in recruitment of leukocytes to the site of vascular injury and enhancement of inflammatory processes. Recently, some studies have revealed that anthocyanin supplementation significantly reduces the serum lipid [69] and pro-inflammatory cytokine levels [70–72], consistent with the effects of anthocyanins observed in our previous studies [32, 35]. In the present study, we first revealed that the reductions in the platelet chemokine levels caused by anthocyanins were positively correlated with the changes in the inflammatory marker and blood lipid levels. In particular, the decrease in the level of CXCL7, which is only secreted by platelets, was positively correlated with the decreases in the LDL-C, hsCRP and IL-1β levels. These findings indicate a potential mechanism by which anthocyanins exert protective effects on the cardiovascular system, achieved through the comprehensive regulation of platelet chemokines, lipid metabolism and inflammation, in which platelet chemokines may play pivotal roles. Selective interference with these functional interactions of platelet chemokines may thus enable the development of customized treatments for specific inflammatory conditions associated with CVDs.

Chemokines interact with their target cells by binding to G protein-coupled receptors to initiate inflammatory responses [73]. Both chemokines and their receptors are the key mediators of inflammatory processes regulating leukocyte extravasation and directional migration to vascular lesions [74–76]. We suspected that chemokine receptors might be among the targets of anthocyanins. Based on this hypothesis, we conducted an in vitro experiment to explore the effects of anthocyanins on platelet chemokine receptors. Interestingly, our primary data showed that anthocyanins significantly inhibited the expression of CXCR2, CXCR4 and CCR5 on leukocytes and platelets (unpublished data). These results indicate that inhibition of the corresponding chemokine receptors may be one of the mechanisms of anthocyanins in reducing inflammatory cell recruitment. In addition, some studies have shown that platelet chemokines promote the recruitment of inflammatory cells to injured tissues by forming heterocomplexes with molecules released from neutrophils and other cells, such as HNP1-CCL5 [77], HMGB1-CXCL12 [78], and CXCL4-CCL5 [79]. Hence, selective disruption of the formation of these heterocomplexes may represent a novel mechanism of action of anthocyanins in weakening the interactions between platelets and other inflammatory cells. Further studies are required to investigate the mechanisms underlying the effects of anthocyanins.

## Conclusions

In conclusion, the results of this study suggest that platelet chemokines may be important targets of anthocyanins in prevention of the development of early atherosclerosis. Anthocyanins exert their cardiovascular protective effects by influencing platelet chemokines, blood lipids and the inflammatory response.

## Abbreviations

ENA-78: Epithelial neutrophil-activating peptide
GRO-α: Growth-regulated oncogene-α
HDL-C: High-density lipoprotein-cholesterol
HsCRP: High-sensitivity C-reactive protein
HUVECs: Activated human umbilical vein endothelial cells
IL-1β: Interleukin-1β
IL-8: Interleukin-8
LDL-C: Low-density lipoprotein-cholesterol
MCP-1: Monocyte chemotactic protein-1
MIF: Macrophage migration inhibitory factor
NAP-2: Neutrophil-activating peptide-2
ox-LDL: Oxidized low density lipoprotein
PAI-1: Human plasminogen activator inhibitor1
SDF-1α: Stromal cell-derived factor 1α
sP-selectin: Soluble P-selectin
TNF-α: Tumornecrosis factor-α

## References

[1] Moore KJ, Sheedy FJ, Fisher EA (2013). Macrophages in atherosclerosis: a dynamic balance. Nat Rev Immunol.

[2] Ridker PM, Danielson E, Fonseca FA, Genest J, Gotto AM, Kastelein JJ (2009). Reduction in C-reactive protein and LDL cholesterol and cardiovascular event rates after initiation of rosuvastatin: a prospective study of the JUPITER trial. Lancet.

[3] Zhang D, Jiang X, Fang P, Yan Y, Song J, Gupta S (2009). Hyperhomocysteinemia promotes inflammatory monocyte generation and accelerates atherosclerosis in transgenic cystathionine beta-synthase-deficient mice. Circulation.

[4] Wang N, Tall AR (2016). Cholesterol in platelet biogenesis and activation. Blood.

[5] Drechsler M, Duchene J, Soehnlein O (2015). Chemokines control mobilization, recruitment, and fate of monocytes in atherosclerosis. Arterioscler Thromb Vasc Biol.

[6] Vajen T, Mause SF, Koenen RR (2015). Microvesicles from platelets: novel drivers of vascular inflammation. Thromb Haemost.

[7] Herter JM, Rossaint J, Zarbock A (2014). Platelets in inflammation and immunity. J Thromb Haemost.

[8] French PA (2009). Platelet functions beyond hemostasis, antiplatelet therapies in high-risk patient subgroups: the fourth annual platelet colloquium. J Thromb Thrombolysis.

[9] Morrell CN, Aggrey AA, Chapman LM, Modjeski KL (2014). Emerging roles for platelets as immune and inflammatory cells. Blood.

[10] Rosenson RS, Brewer HB, Ansell BJ, Barter P, Chapman MJ, Heinecke JW (2016). Dysfunctional HDL and atherosclerotic cardiovascular disease. Nat Rev Cardiol.

[11] Jin F, Hagemann N, Schäfer ST, Brockmeier U, Zechariah A, Hermann DM (2013). SDF-1 restores angiogenesis synergistically with VEGF upon LDL exposure despite CXCR4 internalization and degradation. Cardiovasc Res.

[12] Larsen SB, Grove EL, Würtz M, Neergaard-Petersen S, Hvas AM, Kristensen SD (2015). The influence of low-grade inflammation on platelets in patients with stable coronary artery disease. Thromb Haemost.

[13] Rainger G, Chimen M, Harrison MJ, Yates CM, Harrison P, Watson SP (2015). The role of platelets in the recruitment of leukocytes during vascular disease. Platelets.

[14] von Hundelshausen P, Schmitt MMN (2014). Platelets and their chemokines in atherosclerosis—clinical applications. Front Physiol.

[15] Drechsler M, Megens RT, van Zandvoort M, Weber C, Soehnlein O (2010). Hyperlipidemia-triggered neutrophilia promotes early atherosclerosis. Circulation.

[16] Li X, Zhu M, Penfold ME, Koenen RR, Thiemann A, Heyll K (2014). Activation of CXCR7 limits atherosclerosis and improves hyperlipidemia by increasing cholesterol uptake in adipose tissue. Circulation.

[17] Yang Z, Zhang Z, Wen J, Wang X, Lu B, Yang Z (2010). Elevated serum Chemokine CXC ligand 5 Levels Are associated with hypercholesterolemia but not a worsening of insulin resistance in Chinese people. J Clin Endocrinol Metab.

[18] Karshovska E, Zhao Z, Blanchet X, Schmitt MM, Bidzhekov K, Soehnlein O (2015). Hyperreactivity of junctional adhesion molecule A-Deficient platelets accelerates atherosclerosis in hyperlipidemic mice. Circ Res.

[19] Clarke MC, Talib S, Figg NL, Bennett MR (2010). Vascular smooth muscle cell apoptosis induces interleukin-1-directed inflammation: effects of hyperlipidemia-mediated inhibition of phagocytosis. Circ Res.

[20] Flad HD, Brandt E (2010). Platelet-derived chemokines: pathophysiology and therapeutic aspects. Cell Mol Life Sci.

[21] Wallace TC (2011). Anthocyanins in cardiovascular disease. Adv Nutr.

[22] Jang HH, Park MY, Kim HW, Lee YM, Hwang KA, Park JH (2012). Black rice (Oryza sativa L.) extract attenuates hepatic steatosis in C57BL/6 J mice fed a high-fat diet via fatty acid oxidation. Nutr Metab (Lond).

[23] Giampieri F, Forbes-Hernandez TY, Gasparrini M, Alvarez-Suarez JM, Afrin S, Bompadre S (2015). Strawberry as a health promoter: an evidence based review. Food Funct.

[24] Thompson K, Pederick W, Santhakumar AB (2016). Anthocyanins in obesity-associated thrombogenesis: a review of the potential mechanism of action. Food Funct.

[25] Weh KM, Aiyer HS, Howell AB, Kresty LA (2016). Cranberry proanthocyanidins modulate reactive oxygen species in Barrett’s and esophageal adenocarcinoma cell lines. J Berry Res.

[26] Afrin S, Gasparrini M, Forbes-Hernandez TY, Reboredo-Rodriguez P, Mezzetti B, Varela-López A (2016). Promising health benefits of the strawberry: a focus on clinical studies. J Agric Food Chem.

[27] Edirisinghe I, Burton-Freeman B (2016). Anti-diabetic actions of Berry polyphenols - review on proposed mechanisms of action. J Berry Res.

[28] Luo T, Miranda-Garcia O, Adamson A, Sasaki G, Shay NF (2016). Development of obesity is reduced in high-fat fed mice fed whole raspberries, raspberry juice concentrate, and a combination of the raspberry phytochemicals ellagic acid and raspberry ketone. J Berry Res.

[29] Alvarez-Suarez JM, Giampieri F, Tulipani S, Casoli T, Di Stefano G, González-Paramás AM (2014). One-month strawberry-rich anthocyanin supplementation ameliorates cardiovascular risk, oxidative stress markers and platelet activation in humans. J Nutr Biochem.

[30] Zhu Y, Huang X, Zhang Y, Wang Y, Liu Y, Sun R (2014). Anthocyanin supplementation improves HDL-Associated Paraoxonase 1 activity and enhances cholesterol efflux capacity in subjects with hypercholesterolemia. J Clin Endocrinol Metab.

[31] Zhu Y, Xia M, Yang Y, Liu F, Li Z, Hao Y (2011). Purified anthocyanin supplementation improves endothelial function via NO-cGMP activation in hypercholesterolemic individuals. Clin Chem.

[32] Zhu Y, Ling W, Guo H, Song F, Ye Q, Zou T (2013). Anti-inflammatory effect of purified dietary anthocyanin in adults with hypercholesterolemia: A randomized controlled trial. Nutr Metab Cardiovasc Dis.

[33] Yang Y, Andrews MC, Hu Y, Wang D, Qin Y, Zhu Y (2011). Anthocyanin extract from Black Rice significantly ameliorates platelet hyperactivity and Hypertriglyceridemia in Dyslipidemic Rats induced by high fat diets. J Agric Food Chem.

[34] Yang Y, Shi Z, Reheman A, Jin JW, Li C, Wang Y (2012). Plant Food delphinidin-3-glucoside significantly inhibits platelet activation and thrombosis: novel protective roles against cardiovascular diseases. PLOS One.

[35] Song F, Zhu Y, Shi Z, Tian J, Deng X, Ren J (2014). Plant food anthocyanins inhibit platelet granule secretion in hypercholesterolaemia: involving the signalling pathway of PI3K-Akt. ThrombHaemost.

[36] Weber C (2005). Platelets and Chemokines in atherosclerosis: partners in crime. Circ Res.

[37] Koenen RR. The prowess of platelets in immunity and inflammation. ThrombHaemost. 2016; doi:10.1160/TH16-04-0300 10.1160/TH16-04-0300 27384503

[38] Ghasemzadeh M, Kaplan ZS, Alwis I, Schoenwaelder SM, Ashworth KJ, Westein E (2013). The CXCR1/2 ligand NAP-2 promotes directed intravascular leukocyte migration through platelet thrombi. Blood.

[39] Wang XZ, Liu LW, Du XM, Gu RX, Sun ZJ (2015). CXCL5 is associated with the increased risk of coronary artery disease. Coron Artery Dis.

[40] Dai C, Yao X, Keeran KJ, Zywicke GJ, Qu X, Yu ZX (2012). Apolipoprotein A-I attenuates ovalbumin-induced neutrophilic airway inflammation via a granulocyte colony-stimulating factor-dependent mechanism. Am J Respir Cell Mol Biol.

[41] Mei J, Liu Y, Dai N, Hoffmann C, Hudock KM, Zhang P (2012). Cxcr2 and Cxcl5 regulate the IL-17/G-CSF axis and neutrophil homeostasis in mice. J Clin Invest.

[42] Segers VF, Revin V, Wu W, Qiu H, Yan Z, Lee RT (2011). Protease-resistant stromal cell-derived factor-1 for the treatment of experimental peripheral artery disease. Circulation.

[43] Ahmed S, Pakozdi A, Koch AE (2006). Regulation of interleukin-1beta-induced chemokine production and matrix metalloproteinase 2 activation by epigallocatechin-3-gallate in rheumatoid arthritis synovial fibroblasts. Arthritis Rheum.

[44] Lee JY, Park W (2015). Anti-inflammatory effect of Wogonin on RAW 264.7 mouse macrophages induced with polyinosinic-polycytidylic Acid. Molecules.

[45] Yilma AN, Singh SR, Morici L, Dennis VA (2013). Flavonoid Naringenin: A potential immunomodulator for Chlamydia trachomatis inflammation. Mediators Inflamm.

[46] Souto FO, Zarpelon AC, Staurengo-Ferrari L, Fattori V, Casagrande R, Fonseca MJ (2011). Quercetin reduces neutrophil recruitment induced by CXCL8, LTB4, and fMLP: inhibition of actin polymerization. J Nat Prod.

[47] Ghasemzadeh M, Hosseini E (2013). Platelet-leukocyte crosstalk: linking proinflammatory responses to procoagulant state. Thromb Res.

[48] Habets KL, Huizinga TW, Toes RE (2013). Platelets and autoimmunity. Eur J Clin Invest.

[49] Karlsen A, Retterstøl L, Laake P, Paur I, Bøhn SK, Sandvik L (2007). Anthocyanins inhibit nuclear factor-kappaB activation in monocytes and reduce plasma concentrations of pro-inflammatory mediators in healthy adults. J Nutr.

[50] Kuntz S, Asseburg H, Dold S, Römpp A, Fröhling B, Kunz C (2015). Inhibition of low-grade inflammation by anthocyanins from grape extract in an in vitro epithelial-endothelial co-culture model. Food Funct.

[51] Farrell N, Norris G, Lee SG, Chun OK, Blesso CN (2015). Anthocyanin-rich black elderberry extract improves markers of HDL function and reduces aortic cholesterol in hyperlipidemic mice. Food Funct.

[52] Garcia-Alonso M, Minihane AM, Rimbach G, Rivas-Gonzalo JC, de Pascual-Teresa S (2009). Red wine anthocyanins are rapidly absorbed in humans and affect monocyte chemoattractant protein 1 levels and antioxidant capacity of plasma. J Nutr Biochem.

[53] Björk C, Wilhelm U, Mandrup S, Larsen BD, Bordoni A, Hedén P (2016). Effects of selected bioactive food compounds on human white adipocyte function. Nutr Metab(Lond).

[54] Kuntz S, Kunz C, Herrmann J, Borsch CH, Abel G, Fröhling B (2014). Anthocyanins from fruit juices improve the antioxidant status of healthy young female volunteers without affecting anti-inflammatory parameters: results from the randomised, double-blind, placebo-controlled, cross-over ANTHONIA (ANTHOcyanins in Nutrition Investigation Alliance) study. Br J Nutr.

[55] Karshovska E, Weber C, von Hundelshausen P (2013). Platelet chemokines in health and disease. Thromb Haemost.

[56] Bruserud Ø (2013). Bidirectional crosstalk between platelets and monocytes initiated by toll-like receptor: an important step in the early defense against fungal infections?. Platelets.

[57] Morrison MC, Liang W, Mulder P, Verschuren L, Pieterman E, Toet K (2015). Mirtoselect, an anthocyanin-rich bilberry extract, attenuates non-alcoholic steatohepatitis and associated fibrosis in ApoE(∗)3Leiden mice. J Hepatol.

[58] Jiang Z, Chen C, Xie W, Wang M, Wang W, Zhang X (2016). Anthocyanins attenuate alcohol-induced hepatic injury by inhibiting pro-inflammation signaling. Nat Prod Res.

[59] Zhao R, Le K, Li W, Ren S, Moghadasian MH, Beta T (2014). Effects of Saskatoon berry powder on monocyte adhesion to vascular wall of leptin receptor-deficient diabetic mice. J Nutr Biochem.

[60] Lamy S, Lafleur R, Bédard V, Moghrabi A, Barrette S, Gingras D (2007). Anthocyanidins inhibit migration of glioblastoma cells: structure-activity relationship and involvement of the plasminolytic system. J Cell Biochem.

[61] Broekhuizen LN, Van Wijk DF, Vink H, Stalmach A, Crozier A, Hutten BA (2011). Reduction of monocyte chemoattractant protein 1 and macrophage migration inhibitory factor by a polyphenol-rich extract in subjects with clustered cardiometabolic risk factors. Br J Nutr.

[62] Farrell NJ, Norris GH, Ryan J, Porter CM, Jiang C, Blesso CN (2015). Black elderberry extract attenuates inflammation and metabolic dysfunction in diet-induced obese mice. Br J Nutr.

[63] Gomes AL, Carvalho T, Serpa J, Torre C, Dias S (2010). Hypercholesterolemia promotes bone marrow cell mobilization by perturbing the SDF-1:CXCR4 axis. Blood.

[64] Badrnya S, Butler LM, Söderberg-Naucler C, Volf I, Assinger A (2012). Platelets directly enhance neutrophil transmigration in response to oxidised low-density lipoprotein. Thromb Haemost.

[65] Holm T, Damås JK, Holven K, Nordøy I, Brosstad FR, Ueland T (2003). CXC-chemokines in coronary artery disease: possible pathogenicrole of interactions between oxidized low-density lipoprotein, platelets and peripheral blood mononuclear cells. J Thromb Haemost.

[66] Nassar T, Sachais BS, Akkawi S, Kowalska MA, Bdeir K, Leitersdorf E (2003). Platelet factor 4 enhances the binding of oxidized low-density lipoprotein to vascular wall cells. J Biol Chem.

[67] Wiesner P, Tafelmeier M, Chittka D, Choi SH, Zhang L, Byun YS (2013). MCP-1 binds to oxidized LDL and is carried by lipoprotein(a) in human plasma. J Lipid Res.

[68] Bersinger NA, Günthert AR, McKinnon B, Johann S, Mueller MD (2011). Dose-response effect of interleukin (IL)-1β, tumour necrosis factor (TNF)-α, and interferon-γ on the in vitro production of epithelial neutrophil activating peptide-78 (ENA-78), IL-8, and IL-6 by human endometrial stromal cells. Arch Gynecol Obstet.

[69] Liu C, Sun J, Lu Y, Bo Y (2016). Effects of anthocyanin on serum lipids in dyslipidemia patients: a systematic review and meta-analysis. PLOS One.

[70] Vendrame S, Tsakiroglou P, Kristo AS, Schuschke DA, Klimis-Zacas D (2016). Wild blueberry consumption attenuates local inflammation in the perivascular adipose tissue of obese Zucker rats. Appl Physiol Nutr Metab.

[71] Bhawamai S, Lin S, Hou Y, Chen Y (2016). Thermal cooking changes the profile of phenolic compounds, but does not attenuate the anti-inflammatory activities of black rice. Food Nutr Res.

[72] Shukitt-Hale B, Kelly M, Bielinski D, Fisher D (2016). Tart cherry extracts reduce inflammatory and oxidative stress signaling in microglial cells. Antioxidants.

[73] Sozzani S, Del Prete A, Bonecchi R, Locati M (2015). Chemokines as effector and target molecules in vascular biology. Cardiovasc Res.

[74] Bonecchi R, Graham GJ (2016). Atypical Chemokine Receptors and Their Roles in the Resolution of the Inflammatory Response. Front Immunol.

[75] Li J, McArdle S, Gholami A, Kimura T, Wolf D, Gerhardt T (2016). CCR5 + T-bet + FoxP3+ Effector CD4 T Cells Drive Atherosclerosis. Circ Res.

[76] Wang L, Zhao X, Cui W, Ma Y, Ren H, Zhou X, et al. Genetic and Pharmacologic Inhibition of the Chemokine Receptor CXCR2 Prevents Experimental Hypertension and Vascular Dysfunction. Circulation. 2016; doi: 10.1161/CIRCULATIONAHA.115.020754 10.1161/CIRCULATIONAHA.115.020754 PMC5084654 27678262

[77] Alard JE, Ortega-Gomez A, Wichapong K, Bongiovanni D, Horckmans M, Megens RT (2015). Recruitment of classical monocytes can be inhibited by disturbing heteromers of neutrophil HNP1 and platelet CCL5. Sci Transl Med.

[78] Schiraldi M, Raucci A, Muñoz LM, Livoti E, Celona B, Venereau E (2012). HMGB1 promotes recruitment of inflammatory cells to damaged tissues by forming a complex with CXCL12 and signaling via CXCR4. J Exp Med.

[79] Koenen RR, von Hundelshausen P, Nesmelova IV, Zernecke A, Liehn EA, Sarabi A (2009). Disrupting functional interactions between platelet chemokines inhibits atherosclerosis in hyperlipidemic mice. Nat Med.

